# Postprocessing Algorithm for Driving Conventional Scanning Tunneling Microscope at Fast Scan Rates

**DOI:** 10.1155/2017/1097142

**Published:** 2017-11-20

**Authors:** Hao Zhang, Xianqi Li, Yunmei Chen, Jewook Park, An-Ping Li, X.-G. Zhang

**Affiliations:** ^1^Department of Mathematics, University of Florida, Gainesville, FL 32611, USA; ^2^Center for Nanophase Materials Sciences, Oak Ridge National Laboratory, Oak Ridge, TN 37831-6487, USA; ^3^Center for Artificial Low Dimensional Electronic Systems, Institute for Basic Science (IBS), Pohang 37673, Republic of Korea; ^4^Department of Physics and the Quantum Theory Project, University of Florida, Gainesville, FL 32611, USA

## Abstract

We present an image postprocessing framework for Scanning Tunneling Microscope (STM) to reduce the strong spurious oscillations and scan line noise at fast scan rates and preserve the features, allowing an order of magnitude increase in the scan rate without upgrading the hardware. The proposed method consists of two steps for large scale images and four steps for atomic scale images. For large scale images, we first apply for each line an image registration method to align the forward and backward scans of the same line. In the second step we apply a “rubber band” model which is solved by a novel Constrained Adaptive and Iterative Filtering Algorithm (CIAFA). The numerical results on measurement from copper(111) surface indicate the processed images are comparable in accuracy to data obtained with a slow scan rate, but are free of the scan drift error commonly seen in slow scan data. For atomic scale images, an additional first step to remove line-by-line strong background fluctuations and a fourth step of replacing the postprocessed image by its ranking map as the final atomic resolution image are required. The resulting image restores the lattice image that is nearly undetectable in the original fast scan data.

## 1. Introduction

The invention of the scanning tunneling microscopy (STM) revolutionized the study of nanoscale and atomic scale surface structures and properties [1, 2]. However, STM has rarely been considered a real-time method because of its slow scanning rate compared to most dynamic processes on a surface [3]. This has severely limited its application to the study of most dynamic processes on surfaces such as surface diffusion, phase transitions, self-assembly phenomena, film growth and etching, chemical reactions, and conformational changes of molecules. Raising the scan rate of scanning probes has been the objective of intense research efforts in the past decades [3–5], with most of the efforts focused on hardware improvements. On the other hand, researchers have also applied other techniques to utilize conventional, slow scan STM to study dynamic processes. For example, low-frequency dynamic behavior of a flexible free-standing graphene sheet has been studied using clever postprocessing [6].

The common practice for STM measurement is by bringing the tip close to the sample surface and applying a voltage bias to generate a tunnel current between the tip and sample. The tip is moved across the sample parallel to the surface (in the *xy* plane). Changes in the surface height *z* or in the density of states cause a change in the tunneling current. The change in current with respect to position can be measured itself, or alternatively the height of the tip corresponding to a constant current can be measured. These two modes are called the constant height mode and the constant current mode, respectively. In the constant current mode, feedback electronics adjust the height by a voltage to the piezoelectric height control mechanism. In the constant height mode, the voltage and height are both held constant while the current changes to keep the junction voltage from changing. The constant current mode is usually used in STM because surface features can easily exceed a predefined tip-sample separation (typically 4–7 Å) and can crash the tip in a constant height mode. But the constant current mode is slow, due to more time required by the piezoelectric movements to register the height change. The time to complete a measurement for each pixel position is about 2 msec for a typical equipment and approaches 0.1 msec for a top-of-line setup [3–5].

**Figure 1:**
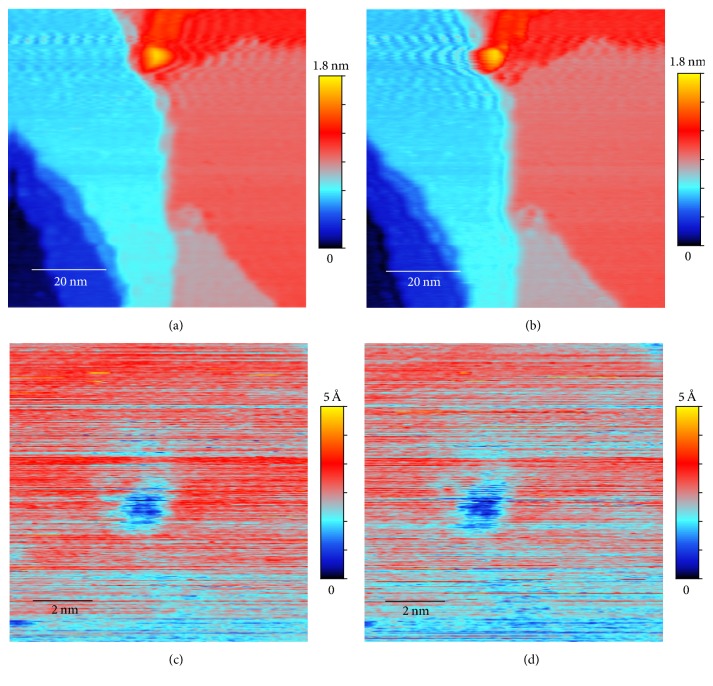
STM data of a single crystal Cu(111) surface obtained with a fast scan rate (0.1 msec per pixel): (a) topography raw data from forward scan (*F*_*o*_), scan area 80 × 80 nm^2^, or effective scanning speed 1.56 *μ*/sec; (b) topography raw data from backward scan (*B*_*o*_); (c) atomic resolution raw data from forward scan, scan area 10 × 10 nm^2^, or effective scanning speed 195 nm/sec; (d) atomic resolution raw data from backward scan. Bright streaks are indications of possible tip crashes.

Conventional STM has a limited scanning speed because of the response time of the electric circuit and the piezoelectric component used to control the movement of the probes. The control of the motion of the tip requires an electric feedback circuit which can generate a resonance at the frequency of the order of 10 kHz. If the scanning speed is too fast large noise appears in the image in the form of a spatial oscillation (Figure 1). However, below a critical scan rate determined by the resonant frequency, such noise usually appears differently in the forward scans and the backward ones. Therefore one can devise an algorithm that exploits the difference between the forward and backward scans to eliminate the oscillatory noise in the image, thus allowing faster scans to be performed.

For atomic resolution images, the error due to a fast scan rate appears to be large intensity fluctuations between scan lines, as shown in Figures 1(c) and 1(d). These large fluctuations are likely caused by “soft” crashes of the tip, an unavoidable problem when the scan rate is driven faster than the response rate of the feedback circuit. However, after each tip crash, the atomic features remain but with a different background intensity. Thus an effective background removal procedure is needed before the forward and backward scan images can be combined to obtain an optimal result.

**Figure 2:**
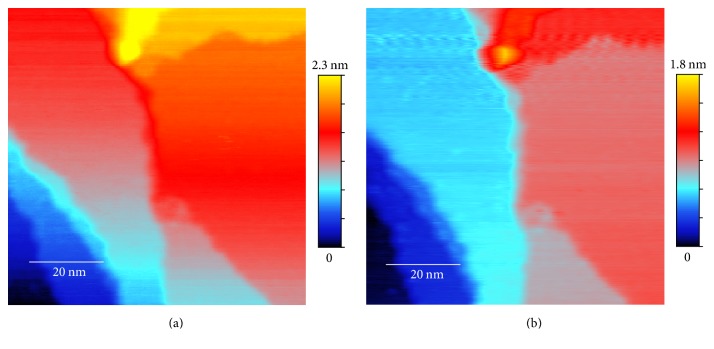
Comparison of processed topography images of the Cu sample in Figures 1(a) and 1(b): (a) slow scan rate (2 msec per pixel) data averaged over forward and backward scans; (b) processed data using the algorithm presented in this paper from the fast scan rate (0.1 msec per pixel) data shown in Figure 1. Scan drift is clearly visible in (a), appearing as a gradual change in the height (represented by the color) of a plateau. There is no visible scan drift in (b).

**Figure 3:**
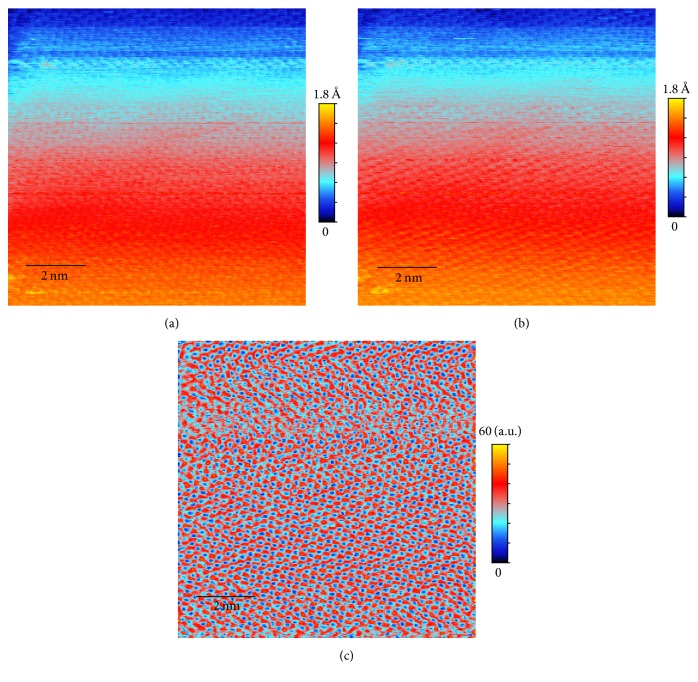
Processed atomic resolution image of the Cu sample from the slow scan data: (a) original forward scan; (b) original backward scan; (c) the ranking map of the postprocessed image as the final atomic resolution image.

**Figure 4:**
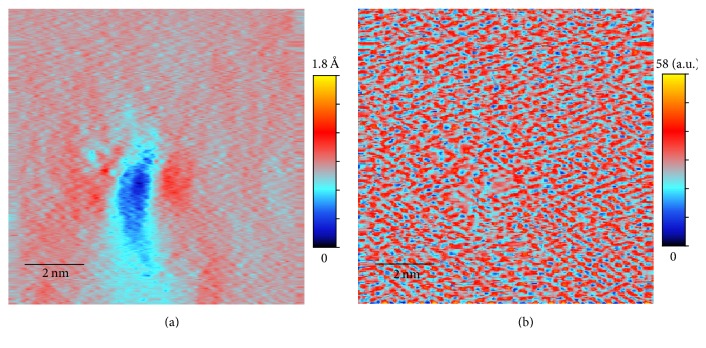
Processed atomic resolution image of the Cu sample from the fast scan data in Figures 1(c) and 1(d): (a) result after background removal, registration, and image restoration steps; (b) the ranking map of the postprocessed image as the final atomic resolution image.

The effectiveness of the method is demonstrated on topography data from a single crystal Cu(111) surface. Cu surface is a prototypical platform for atomic resolution STM studies on electron scattering [7], nanostructure synthesis [8, 9], and catalysis behaviors [10]. The faster scanning will allow revealing dynamic processes on Cu surface, such as molecular self-assembly [11], surface diffusion [12], and chemical reaction [13]. We will show that, after processing using the algorithm presented in this paper, the obtained topography image from a fast scan rate of 0.1 msec per pixel (effective scanning speed 1.56 *μ*/sec for scan area 80 × 80 nm^2^) nearly matches the quality of the image of the same sample from a slow scan rate of 2 msec per pixel and also eliminates the scan drift that is apparent in the slow scan image (Figure 2). For atomic resolution data, our method is able to greatly improve the accuracy of a slow scan data (Figure 3) and to recover atomic images (Figure 4) from a fast scan data with a scan rate also at 0.1 msec per pixel (effective scanning speed 195 nm/sec for scan area 10 × 10 nm^2^) where the raw image is overwhelmed by noise and tip crash streaks (see Figure 1).

The paper is organized as follows: we introduce our methods of background removal in Section 2.1 and signal registration in Section 2.2, followed by the proposed signal restoration method in Section 2.3 and atomic image extraction method in Section 2.4. The description of the STM experimental setup, sample preparation, and results of our method applied on a data set with fast scan speed and comparison to data with slow scan speed are presented in Section 3. Concluding remarks are given in Section 4.

## 2. Method

### 2.1. Line-by-Line Background Removal

The intensity fluctuation in the atomic resolution images seriously degrades the image quality and impedes the registration and image restoration method that we will apply later. Because the image intensity varies strongly from one scan line to the next, but stays nearly constant over long segments within each line, this fluctuation can be treated as a background for each scan line. Therefore, the first step in processing the atomic resolution images is to remove this background in a line-by-line manner.

The forward and the backward scans are represented by *F*_*a*_ ∈ *ℝ*^*m*×*n*^ and *B*_*a*_ ∈ *ℝ*^*m*×*n*^, respectively. Consider one of the images, say *F*_*a*_, whose *i*th line is represented by *f* ∈ *ℝ*^1×*n*^. The background is considered a slow varying function along the line. We wish to remove the background and keep the fast varying features. To achieve this, we estimate the value of the background at point *x*, *g*_*x*_, by a weighted least mean-square linear fit to a segment of the line centered at *x* with length *L*:

(1) minax,gx ∑x′=x−L/2x+L/2fx′−axx′−x−gx2wx′−x,

where

(2) $$\begin{array}{c} w\left(\;x^{\prime }-x\;\right)=\frac{L^{2}}{L^{2}+4{\left(x^{\prime }-x\right)}^{2}}. \end{array}$$

The weight *w*(*x*′ − *x*) is to reduce the impact of noise in the data. It is a numerically efficient alternative to robust least mean-square fits. The corrected image is given by

(3) $$\begin{array}{c} \bar{f}\left(\;x\;\right)=f\left(\;x\;\right)-g_{x}, \end{array}$$

for each *x*. We found numerically $\sum_{x}\bar{f}(x)\ll \sum_{x}|\bar{f}(x)|$. This means that on average the corrected image intensity is close to zero. This removes most of the background and brings all lines to about the same intensity (close to zero).

An additional minor improvement to the background removal process is to remove the small remnant slope at the two edges by fitting a small part of the line at two ends to a linear background with slopes of the same magnitude but opposite sign (so that the two lines meet at the same height in the middle) and removing this background from the corresponding halves of the line. This step makes the intensity somewhat more uniform across the whole line.

The value of *L* is determined by searching for the maximum correlation coefficient after image registration, a process that we will describe below.

The effect of the background removal step using linear regression allows the subsequent two steps to be carried out which produced Figure 4(a), whereas without this step the steps described in the following two subsections are ineffective to process the original image in Figure 1 due to the overwhelming noise.

### 2.2. Image Registration

**Figure 5:**
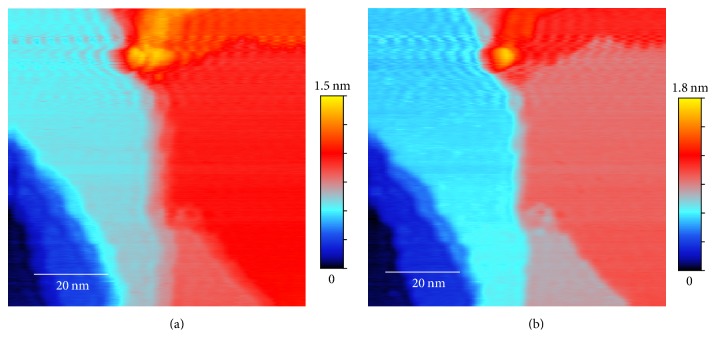
Average of the forward and backward scans in Figure 1: (a) average of the unprocessed data, (1/2)(*F*_*o*_ + *B*_*o*_); (b) average of the aligned data, (1/2)(*F*_*a*_ + *B*_*a*_).

**Figure 6:**
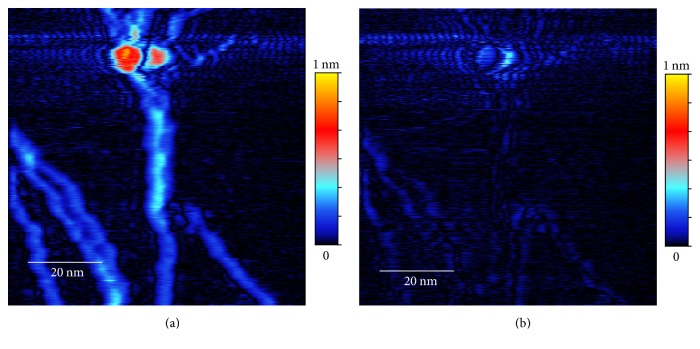
Difference of the forward and backward scans in Figure 1: (a) difference between the unprocessed data (|*F*_*o*_ − *B*_*o*_|); (b) difference between the aligned data (|*F*_*a*_ − *B*_*a*_|).

Our goal is to combine data from forward and backward scans to obtain a more accurate image. Because there is always a small registration difference between the forward and backward scans, a simple combination of the unprocessed data (e.g., a simple average) will yield a blurred image (Figure 5(a)). Indeed, the difference between the forward and backward scans is large near the features in the image (Figure 6(a)). Therefore it is necessary to first match the two scans via a process of image registration before combining them.

The method for image registration is improved significantly from the one used in [14]. The main differences are that we now use a new global registration method different than the one used in [14] and that we no longer use a deformation based local registration method. The latter tends to lock onto the noise thus magnifying the effects of noise.

Although the images are two-dimensional, the data is acquired in a line-by-line manner. Therefore the image registration method is applied separately to each line of the forward and the backward scans, *F*_*a*_ and *B*_*a*_. In the registration procedure, each pair of corresponding lines from *F*_*a*_ and *B*_*a*_ are adjusted by a line shift to minimize their difference. The line shift is calculated for the *i*th lines of *F*_*a*_ and *B*_*a*_, which are represented by *f* ∈ *ℝ*^1×*n*^ and *b* ∈ *ℝ*^1×*n*^, respectively, as a minimization problem. The objective is to find a constant *c*, such that *f*(*x* + *c*) is as close to *b*(*x* − *c*) as possible. In [14], the condition is expressed as the following model:

(4) minc∈Z fx+c−bx−c2.

The small searching space for *c* in practice allows one to solve this minimization through a heuristic search. However, this method is susceptible to noise, especially for atomic resolution data in which presence of noise signal may have the same magnitude as images of atoms. Therefore we need a more robust approach to image registration.

The improved approach is to treat the pair of data sets *F*_*a*_ and *B*_*a*_ as linearly correlated data and find the constant *c* such that the correlation coefficient is maximized:

(5) maxc∈Z ∑xfx+c−favgbx−c−bavgfx+c−favg2bx−c−bavg2,

where *f*_avg_ = (1/*N*)∑_*x*_*f*(*x* + *c*) and *b*_avg_ = (1/*N*)∑_*x*_*b*(*x* − *c*), *N* is the number of points in each line, and $\sqrt{{\left\|f\left(x\right)\right\|}^{2}}$ denotes the quadratic mean of *f*(*x*).

The benefit of image registration is clearly visible in Figure 5(b), which shows the simple average of the aligned data *F*_*a*_ and *B*_*a*_. The average image is much sharper compared to Figure 5(a). The difference between the aligned images, |*F*_*a*_ − *B*_*a*_| shown in Figure 6(a), is also greatly reduced compared to that of the unprocessed data (Figure 6(b)). However, even with the aligned data, the spurious oscillations still exist. In fact, in the best case scenario, a simple average would only reduce the spurious oscillations by about half. A better algorithm is needed to eliminate these oscillations, as we will present next.

### 2.3. Image Restoration

**Figure 7:**
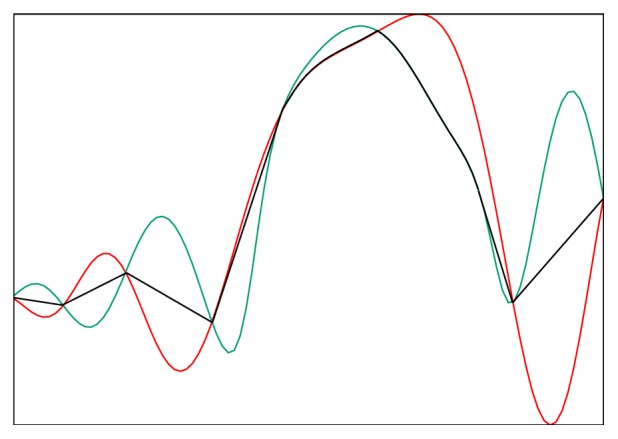
Illustration of the rubber band method using simulated data. The red and green curves are simulated forward and backward scan signals, respectively. A rubber band (black) is inserted between the two curves and pulled tight, yielding the final approximation of the true image.

Using the aligned forward scan *F*_*a*_ and backward scan *B*_*a*_, we wish to find an approximate image that is as close to the true image *S* as possible. Our main goal is to eliminate any noise and spurious signals in the image, while keeping all features in the image. We consider any feature that exists only in one (forward or backward) scan but is not matched in the other scan as spurious and must be removed. To remove such spurious signals, we propose a “rubber band” model, in which a “rubber band” is inserted between the curves of the two scanned signals and is pulled tight, as illustrated in Figure 7. The “rubber band” curve is the final image that most closely represents the true signal. Mathematically, let *s* denote one row of *S* while *f* and *b* represent the corresponding rows of *F*_*a*_ and *B*_*a*_. The associated model for this pair of lines is given by

(6) mins ∑p=2nsp−sp−12s.t. sp≤maxfp,bp, sp≥minfp,bp,where  p=1,…,n.

This model can be solved in an efficient manner by repeatedly smoothing a candidate signal inserted between the two curves *F*_*a*_ and *B*_*a*_ via a constrained signal filtering that maintains the filtered value within the bounds set by *F*_*a*_ and *B*_*a*_ until convergence. The existence of the solution is guaranteed as the average of the forward and backward scans satisfies the constraint and we use it as the initial candidate signal. For the filtering process, the value of each point on the line is replaced by the average value of its two nearest neighbors, subject to the constraint that it is bounded by *F*_*a*_ and *B*_*a*_. The algorithm is outlined in Algorithm 1.

In Algorithm 1, the notation *F*(:, *r*) represents the *r*th row of the 2D matrix *F* and the operators min{·, ·} and max{·, ·} generate vectors of element-wise minimum or maximum value of the two input vectors, respectively.

### 2.4. Construction of the Ranking Map

The first three postprocessing steps described above on atomic resolution fast scan data yield images that clearly show surface lattice structure, as in Figure 4(a). However, this image, like most atomic resolution STM images, contains both the information of atomic positions and the topography of the surface. The latter tends to obstruct the visibility of atomic positions. If the topography information is discarded, then one can obtain a higher quality image containing only atomic positions. In this section we will describe such an algorithm.

The first part of this algorithm is to remove all large scale topography information while retaining the local height information that is needed to distinguish the atoms. To achieve this, we denote by *Ω*_*x*,*y*_ the square consisting of *n* × *n* pixels centered at (*x*, *y*) and define a ranking function *R*(*x*, *y*) for *S*(*x*, *y*) on *Ω*_*x*,*y*_ as follows: If *S*(*x*, *y*) is the *i*th smallest one among all *S*(*x*′, *y*′) for (*x*′, *y*′) ∈ *Ω*_*x*,*y*_, define *R*(*x*, *y*) = :*i* − 1. Then, we replace *S*(*x*, *y*) by *R*(*x*, *y*). Clearly, we have

(7) Rx,y=0,if  Sx,y=minx′,y′∈Ωx,ySx′,y′,n2−1,if  Sx,y=maxx′,y′∈Ωx,ySx′,y′,

This ensures that the entire map is ranged between 0 and *n*^2^ − 1. The best value for *n* is the number of pixels that covers more than 1-2 atomic distances.

The ranking map obtained this way is a ragged image. We apply a 2D median filter [15] to obtain a smoothed image.

The idea of using the ranking map for feature enhancement can be viewed as the reverse of ranking based smoothing techniques such as median filtering. However the ranking map algorithm presented here is unique in that it does not merely use the ranking to help determine the image intensity as in the median filtering method. In the ranking map method, the final image intensity is the ranking itself, and the original image is discarded once the ranking map is constructed.

## 3. Experimental Setup and Results

To establish the effectiveness of the algorithm, it is tested on STM topography scans of a clean copper(111) single crystal surface at room temperature (299 K) using a tungsten tip under conventional scan speed (about 2 msec per data point) as well as fast scan speed of 0.1 msec per data point with multiple scans for the same region on the sample surface. The STM scans are acquired by using a variable temperature STM (Omicron) with Nanonis (SPECS) controller in constant current mode. Clean Cu(111) single crystal was prepared by cycles of Ar+ sputtering and postannealing (550°C) in ultrahigh vacuum condition (8 × 10^−11^ Torr). Electrochemically etched tungsten tip was cleaned by in situ electron bombardment heating.

**Figure 8:**
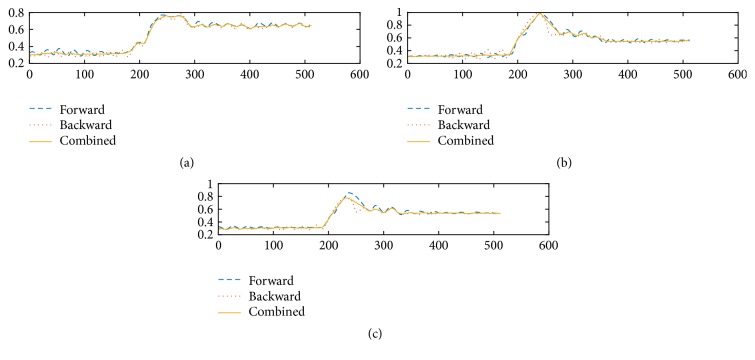
The aligned forward and backward data from the same line of scans: (a) row 46; (b) row 80; (c) row 100.

As already discussed in previous sections, the original unprocessed topography data from a fast scan with a scan rate of 0.1 msec per pixel are shown in Figures 1(a) and 1(b). We can see that both original forward scan and backward scan suffer from significant noise but the patterns of noise are different, making noise elimination possible through combining the two images. The averages and the differences between the original forward and backward scans and the aligned ones after image registration are shown in Figures 5 and 6. The effect of the image restoration algorithm on the experimental image is shown in Figure 8 for three selected rows of the data. The final corrected image is compared to a set of STM scans with slow scan rate on the same surface in Figure 2.

For the atomic resolution fast scan data, the raw image in Figures 1(c) and 1(d) is overwhelmed by noise and has no observable atomic structure. The final processed image (Figure 4(b)), however, displays clearly the surface lattice structure. One can even see the significant lattice strain and disorder due to the proximity of a large defect.

## 4. Conclusion

We have demonstrated an algorithm that can greatly reduce the noise and error of STM images by combining forward and backward scan data in a line-by-line manner. This allows us to push the scan rate for a conventional STM setup to beyond its normal limit, up to 10 times faster. An order of magnitude increase in the scan rate will greatly reduce systematic errors such as the scan drift and environmental noise and will also improve research productivity. Furthermore, this increase represents a first step towards the goal of real-time observation of dynamic processes on surface by STM.

Using the ranking map as the atomic resolution image, as described in this work, is a general algorithm for postprocessing STM images beyond fast scans. Any images obstructed by significant surface topology variations can be treated by this algorithm to yield sharp, atomic resolution results. This algorithm is also useful as an alternative way to enhance image features and can be used as a general tool in image processing technology.

## Figures and Tables

**Algorithm 1:**
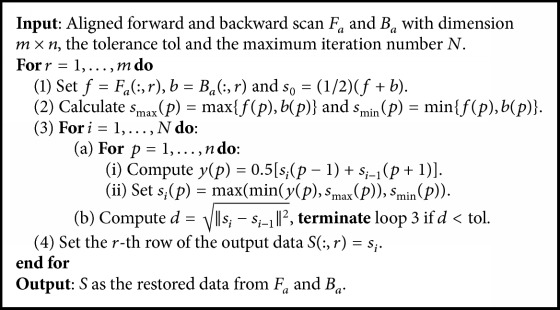
The Constrained Adaptive and Iterative Filtering Algorithm.

## References

[1] Binnig G., Rohrer H. (1982). Scanning Tunneling Microscopy. *Helvetica Physica Acta*.

[2] Tersoff J., Hamann D. R. (1983). Theory and application for the scanning tunneling microscope. *Physical Review Letters*.

[3] Li Q., Lu Q. (2011). Atomic resolution ultrafast scanning tunneling microscope with scan rate breaking the resonant frequency of a quartz tuning fork resonator. *Review of Scientific Instruments*.

[4] Rost M. J., Crama L., Schakel P. (2005). Scanning probe microscopes go video rate and beyond. *Review of Scientific Instruments*.

[5] Esch F., Dri C., Spessot A. (2011). The FAST module: an add-on unit for driving commercial scanning probe microscopes at video rate and beyond. *Review of Scientific Instruments*.

[6] Xu P., Neek-Amal M., Barber S. D. (2014). Unusual ultra-low-frequency fluctuations in freestanding graphene. *Nature Communications*.

[7] Heller E. J., Crommie M. F., Lutz C. P., Eigler D. M. (1994). Scattering and absorption of surface electron waves in quantum corrals. *Nature*.

[8] Stroscio J. A., Celotta R. J. (2004). Controlling the dynamics of a single atom in lateral atom manipulation. *Science*.

[9] Torija M. A., Li A. P., Guan X. C., Plummer E. W., Shen J. (2005). “livea” surface ferromagnetism in Fe nanodots/Cu multilayers on Cu(111). *Physical Review Letters*.

[10] Gawande M. B., Goswami A., Felpin F.-X. (2016). Cu and Cu-based nanoparticles: synthesis and applications in catalysis. *Chemical Reviews*.

[11] Li Q., Owens J. R., Han C. (2013). Self-organized and Cu-coordinated surface linear polymerization. *Scientific Reports*.

[12] Dulot F., Eugène J., Kierren B., Malterre D. (2000). STM-TIP induced surface diffusion of copper on copper (100). *Applied Surface Science*.

[13] Hla S.-W., Meyer G., Rieder K.-H. (2001). Inducing single-molecule chemical reactions with a UHV-STM: A new dimension for nano-science and technology. *ChemPhysChem*.

[14] Zhang H., Li X., Chen Y., Durand C., Li A.-P., Zhang X.-G. (2016). Conductivity map from scanning tunneling potentiometry. *Review of Scientific Instruments*.

[15] Huang T. S., Yang G. J., Tang G. Y. (1979). A Fast Two-Dimensional Median Filtering Algorithm. *IEEE Transactions on Signal Processing*.

